# Supplementary material for: Embryonic signatures of intergenerational epigenetic inheritance across paternal environments and genetic backgrounds
Source: EMBO J. 2025 Sep 26;44(22):6750–81. doi: 10.1038/s44318-025-00556-4 (PMC12624057; doi:10.1038/s44318-025-00556-4)
Supplement: Supplementary file 21 — Expanded View Figures [file 44318_2025_556_MOESM21_ESM.pdf]

Expanded View Figures

Figure EV1. The impact of specific environmental exposures on paternal physiology and F1 blastocysts.

(**A**) Line plot showing growth curves of FVB males (F0 fathers) across CON, nABX and LPHS treatments. Error bars indicate standard deviation of mouse weights at each timepoint. Unpaired t-test; ns: p-value non-significant; *P value < 0.05. (**B**) Kaplan–Meier plot showing male mice's survival during the 6 weeks of indicated environmental exposure. (**C**) Testis to bodyweight ratio. Bars represent the median. (**D**) Representative images of males with open body cavity showing overt physiological responses after 6–7 weeks of indicated treatment. (**E**) Images of full (top) and empty (bottom) male ceca after 6–7 weeks of indicated treatment. (**F**) Representative images of F1 FVB blastocyst embryos generated through IVF. (**F**, **G**) Analysis of F1 blastocyst embryos with Copas Vision Sorter. Inset are images of blastocysts that had different TOF values demonstrating equivalence. (**G**) Scatter plot showing the time of flight (TOF) and Extinction of each blastocyst embryo colour by condition and by batch of experiments. Representative images of blastocysts from both groups are shown. No morphological differences were observed between the groups. Violin plot showing quantification of TOF or extinction. Unpaired t-test; ns: P value non-significant. (**H**) Perimeter, Roughness and mean greyscale of F1 FVB embryos (see 'Methods'). Unpaired t-test; ns: p-value non-significant. (**G**, **H**) [#samples: CON = 80, nABX=73, LPHS = 65]. [Box plots: horizontal black line at the centre is the median. The box extends from the first quartile (Q1: 25th percentile) to the third quartile (Q3: 75th percentile). The vertical black line extends from Q1 − 1.5 × IQR to Q3 + 1.5 × IQR, where IQR = interquartile range. The minima and maxima are not explicitly annotated in these figures.] (**I**) Bar chart depicting efficiency of blastocyst development used for Smart-seq across multiple batches in the FVB background. (**J**) Table with numbers of embryos collected for Smart-seq (top) and passed quality thresholds for Smart-seq bioinformatic analysis (bottom). (**K**) Dot plot showing depth of blastocyst sequencing obtained across batches coloured by paternal condition. (**L**) Grouped bar chart showing number of male and female blastocysts across batches. (**M**) Upset plot showing overlap of DE genes between nABX-derived and LPHS-derived blastocysts. P values are calculated using Fisher's exact test. (**N**) Bubble plot showing gene set enrichment analysis (GSEA) in nABX-derived (left) and LPHS-derived (right) blastocysts. + indicates activated pathways upon paternal treatment, − indicates suppressed pathways upon paternal treatment. (**O**) Principal Component Analysis (PCA) on genes identified to be DE in either nABX-derived or LPHS-derived blastocysts coloured by paternal treatment. Shaded histograms represent relative density of blastocysts along PC1 (top) and PC2 (right).

 © The Author(s)

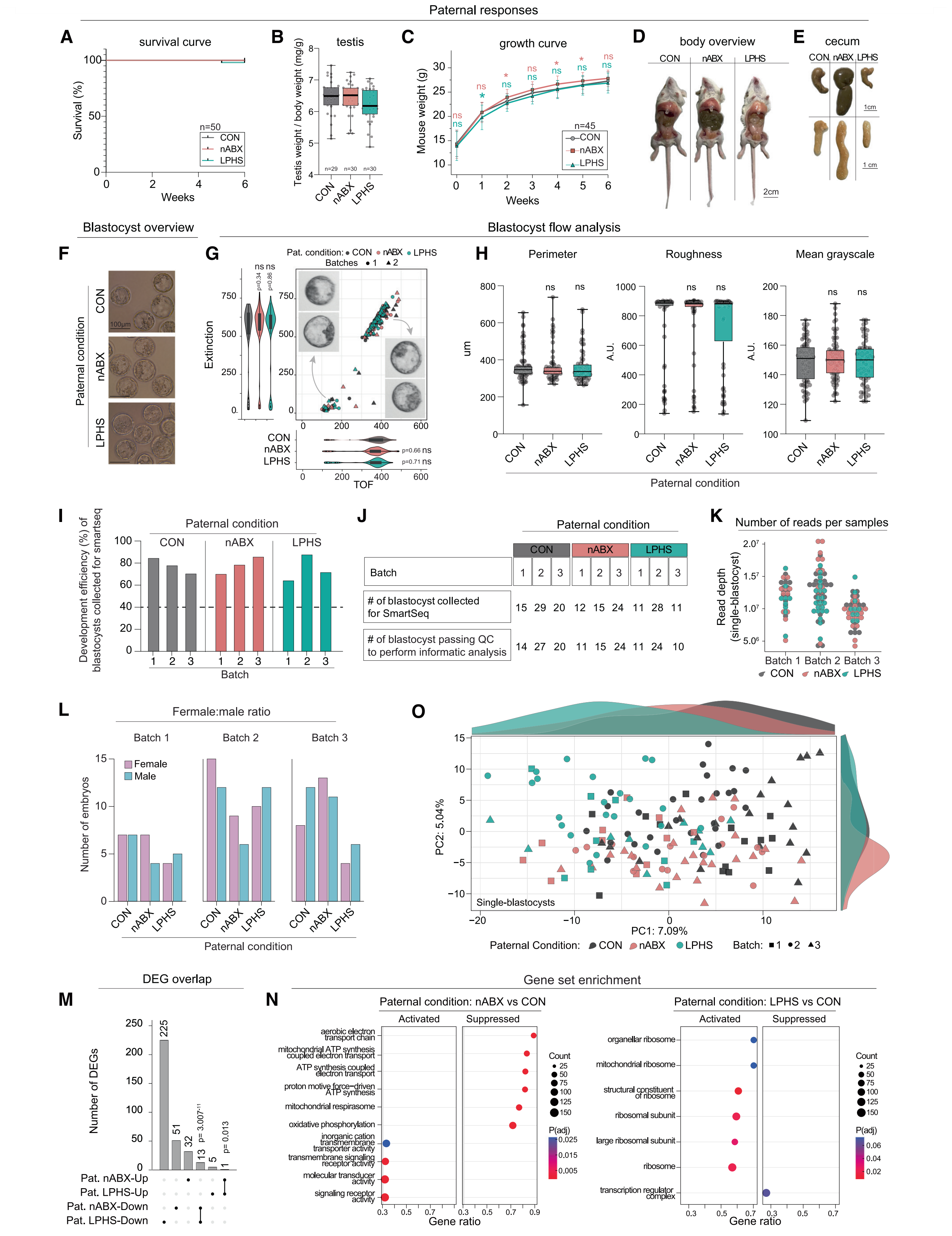
Paternal responses
A
survival curve
Survival (%)
Weeks
n=50
CON
nABX
LPHS
B
testis
Testis weight / body weight (mg/g)
n=29 n=30 n=30
CON nABX LPHS
C
growth curve
Mouse weight (g)
Weeks
n=45
CON
nABX
LPHS
D
body overview
CON nABX LPHS
2cm
E
cecum
CON nABX LPHS
1cm
1 cm
Blastocyst overview
Blastocyst flow analysis
F
Paternal condition
CON
nABX
LPHS
100µm
G
Pat. condition: CON nABX LPHS
Batches 1 2
Extinction
p=0.34 p=0.86
p=0.66 ns
p=0.71 ns
TOF
H
Perimeter
Roughness
Mean grayscale
um
A.U.
A.U.
Paternal condition
I
Paternal condition
CON nABX LPHS
Development efficiency (%) of blastocysts collected for smartseq
Batch
J
Paternal condition
CON nABX LPHS
Batch 1 2 3 1 2 3 1 2 3
# of blastocyst collected for SmartSeq 15 29 20 12 15 24 11 28 11
# of blastocyst passing QC to perform informatic analysis 14 27 20 11 15 24 11 24 10
K
Number of reads per samples
Read depth (single-blastocyst)
Batch 1 Batch 2 Batch 3
CON nABX LPHS
L
Fermale:male ratio
Batch 1 Batch 2 Batch 3
Female
Male
Number of embryos
Paternal condition
O
PC2: 5.04%
PC1: 7.09%
Single-blastocysts
Paternal Condition: CON nABX LPHS Batch: 1 2 3
DEG overlap
M
Number of DEGs
225 51 32 13 5 1
p= 3.007⁻¹¹
p= 0.013
Pat. nABX-Up
Pat. LPHS-Up
Pat. nABX-Down
Pat. LPHS-Down
Gene set enrichment
N
Paternal condition: nABX vs CON
Activated Suppressed
aerobic electron transport chain
mitochondrial ATP synthesis coupled electron transport
ATP synthesis coupled electron transport
proton motive force−driven ATP synthesis
mitochondrial respirasome
oxidative phosphorylation
inorganic cation transmembrane transporter activity
transmembrane signaling receptor activity
molecular transducer activity
signaling receptor activity
Gene ratio
Count
P(adj)
Paternal condition: LPHS vs CON
Activated Suppressed
organellar ribosome
mitochondrial ribosome
structural constituent of ribosome
ribosomal subunit
large ribosomal subunit
ribosome
transcription regulator complex
Gene ratio

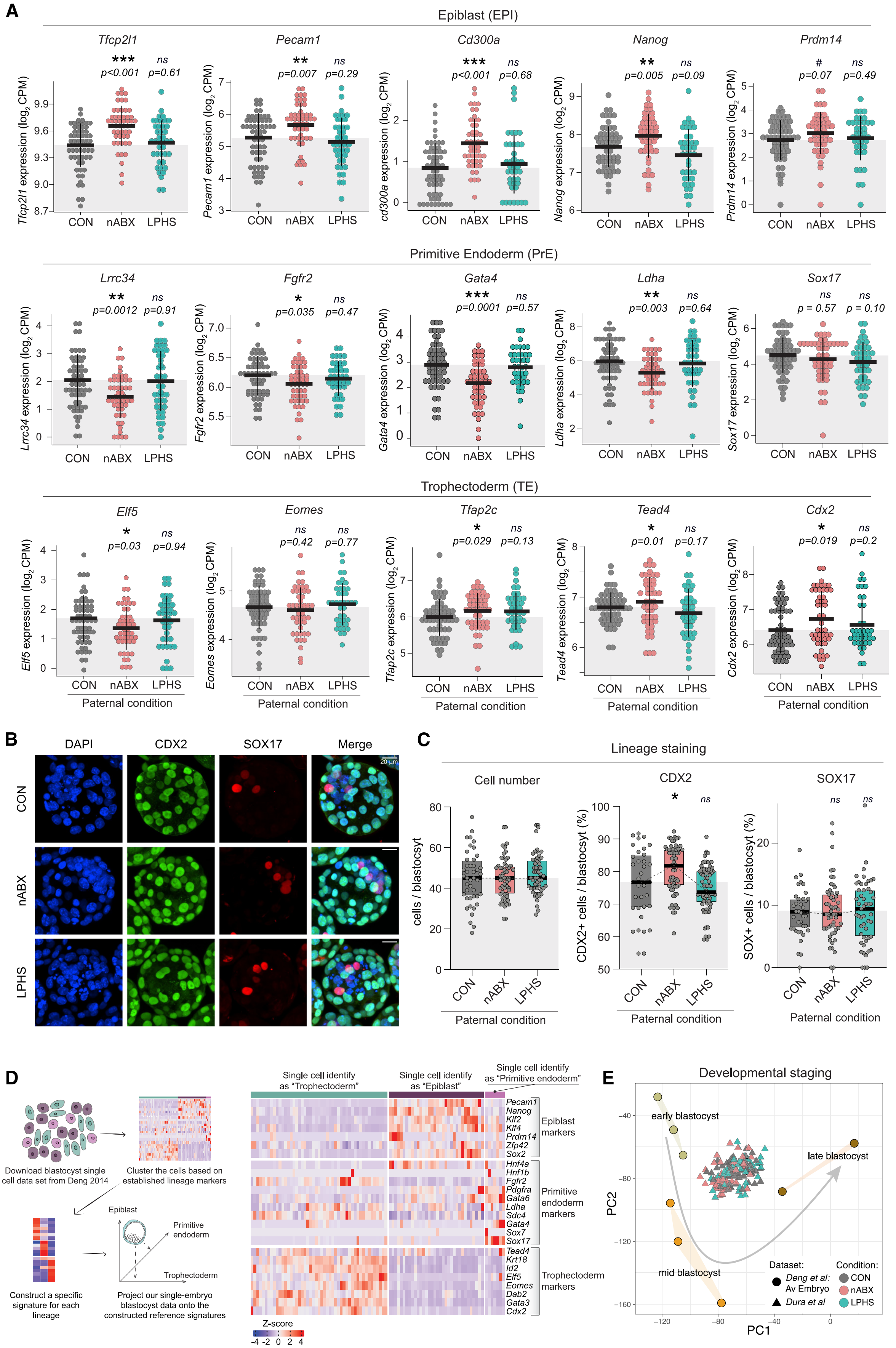
A
Epiblast (EPI)
Tfcp2l1
Pecam1
Cd300a
Nanog
Prdm14
Primitive Endoderm (PrE)
Lrrc34
Fgfr2
Gata4
Ldha
Sox17
Trophectoderm (TE)
Elf5
Eomes
Tfap2c
Tead4
Cdx2
CON nABX LPHS
Paternal condition
B
DAPI
CDX2
SOX17
Merge
C
Lineage staining
Cell number
CDX2
SOX17
D
Download blastocyst single cell data set from Deng 2014
Cluster the cells based on established lineage markers
Construct a specific signature for each lineage
Project our single-embryo blastocyst data onto the constructed reference signatures
Single cell identify as "Trophectoderm"
Single cell identify as "Epiblast"
Single cell identify as "Primitive endoderm"
Epiblast markers
Primitive endoderm markers
Trophectoderm markers
Z-score
E
Developmental staging
early blastocyst
mid blastocyst
late blastocyst
Dataset:
Deng et al: Av Embryo
Dura et al
Condition:
CON
nABX
LPHS
PC1
PC2

 © The Author(s)

Figure EV2. Characterising the IEI lineage-associated impact in F1 blastocysts.

(**A**) Dot plots showing expression of lineage-associated DE genes in F1 blastocysts, driven by at least one paternal condition. Each datapoint is a single blastocyst. Shown are classical and regulatory genes for epiblast, primitive endoderm and trophectoderm lineages. *P values* were computed using a two-tailed Wilcoxon test. [*Tfcp21* nABX *P* value = 6e-06; *Cd300a* nABX *P* value = 5.4e-06]. [#samples: CON = 61, nABX=50, LPHS = 41]. The horizontal black line is the median and the vertical black line extends from Q1 − 1.5 × IQR to Q3 + 1.5 × IQR, where Q1 and Q3 refer to the first and third quartiles respectively and IQR = interquartile range (range of 25th to 75th percentile of the distribution). (**B**) Representative immunofluorescence images of blastocysts sired by control, nABX or LPHS exposed fathers. Staining for CDX2 and SOX17 is shown. (**C**) Quantification of blastocyst cell number and cell lineages as indicated from two independent batches. Each datapoint is an independent blastocyst. %CDX2 *P* value = 0.034. *P* values were computed using a two-tailed unpaired *t* test. (**D**) Schematic explaining the strategy to obtain lineage-specific signatures from published single-cell data (Deng, 2014). (**E**) Projection of our single-blastocyst transcriptomes onto the first two principal components of averaged single-cell transcriptomics data from Deng et al, (2014) (see “Methods”: In silico developmental staging).

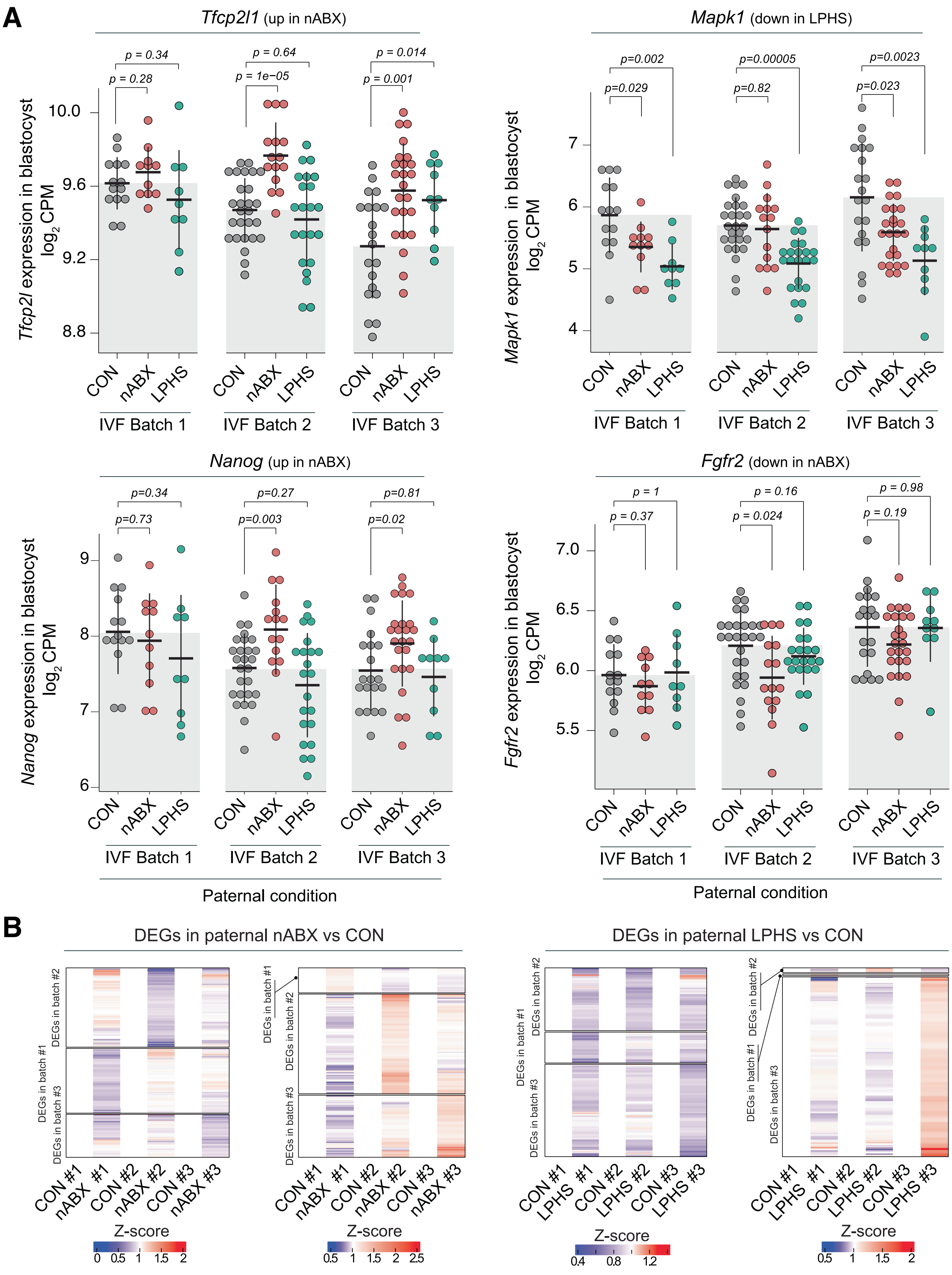
A
Tfcp2l1 (up in nABX)
Tfcp2l expression in blastocyst log2 CPM
p = 0.34
p = 0.28
p = 0.64
p = 1e−05
p = 0.014
p = 0.001
Mapk1 (down in LPHS)
Mapk1 expression in blastocyst log2 CPM
p=0.002
p=0.029
p=0.00005
p=0.82
p=0.0023
p=0.023
Nanog (up in nABX)
Nanog expression in blastocyst log2 CPM
p=0.34
p=0.73
p=0.27
p=0.003
p=0.81
p=0.02
Fgfr2 (down in nABX)
Fgfr2 expression in blastocyst log2 CPM
p = 1
p = 0.37
p = 0.16
p = 0.024
p = 0.98
p = 0.19
CON
nABX
LPHS
IVF Batch 1
IVF Batch 2
IVF Batch 3
Paternal condition
B
DEGs in paternal nABX vs CON
DEGs in paternal LPHS vs CON
DEGs in batch #1
DEGs in batch #2
DEGs in batch #3
CON #1
nABX #1
CON #2
nABX #2
CON #3
nABX #3
LPHS #1
LPHS #2
LPHS #3
Z-score

 © The Author(s)

Figure EV3. Assessing batch-effects in IVF-derived F1 blastocysts.

(**A**) Dot plots showing log expression of genes in in F1 blastocysts derived from the indicated paternal condition, stratified by experimental batch: *Tfcp1l1, Nanog* (epiblast markers), *Mapk1 and Fgfr2 (signalling components)*. Each datapoint indicates a single blastocyst. *P-values* were computed using a two-tailed Wilcoxon test. [#samples Batch 1: CON = 14, nABX=11, LPHS = 9; Batch 2: CON = 27, nABX=15, LPHS = 22; Batch 3: CON = 20, nABX=24, LPHS = 10]. The horizontal black line is the median and the vertical black line extends from Q1 − 1.5 × IQR to Q3 + 1.5 × IQR, where Q1 and Q3 refer to the first and third quartiles respectively and IQR = interquartile range (range of 25th to 75th percentile of the distribution). (**B**) Heatmaps showing fold changes of batch-specific DE genes relative to controls across all batches, split by batch of origin of the DE genes.

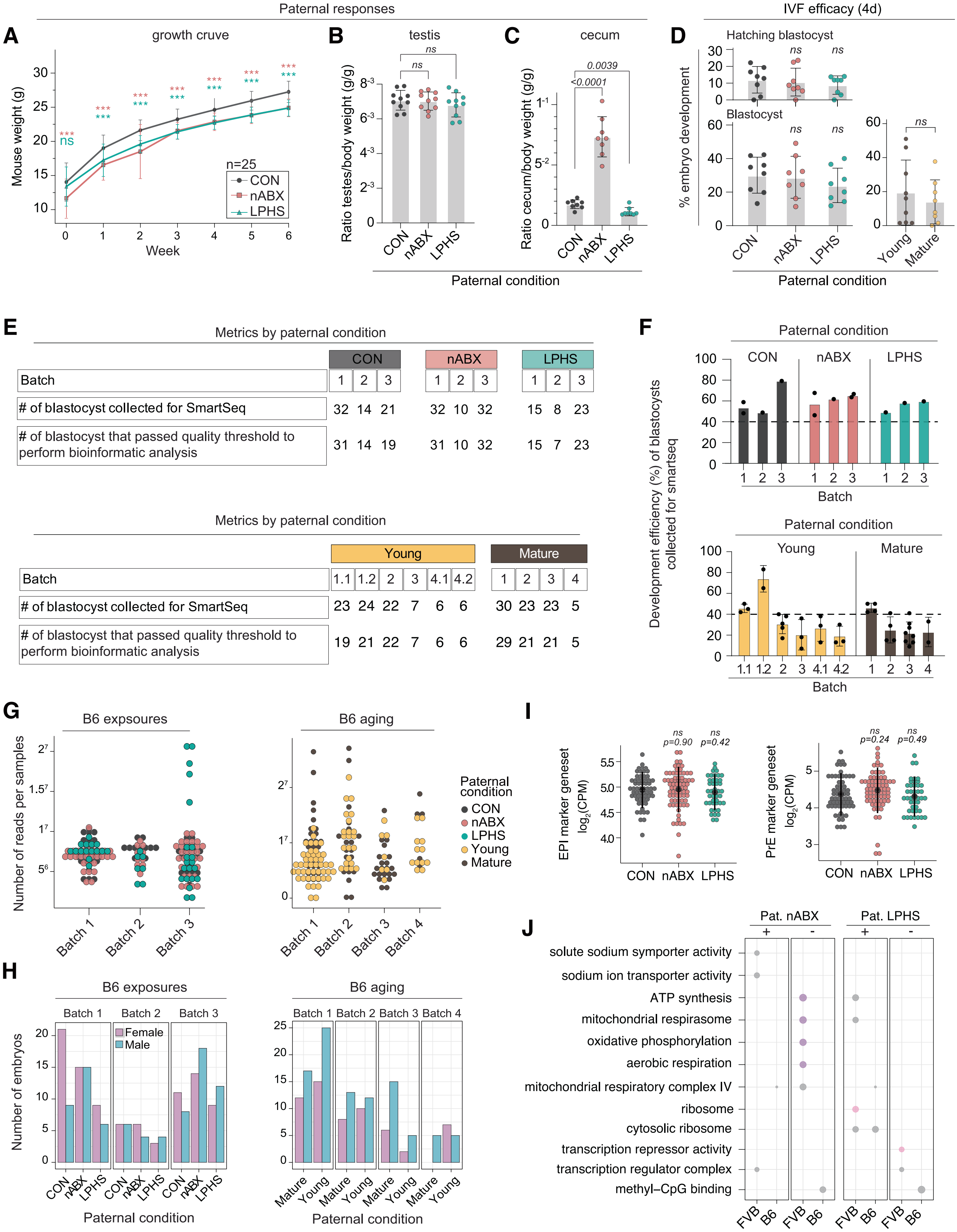
Paternal responses
IVF efficacy (4d)
A
growth cruve
Mouse weight (g)
Week
n=25
CON
nABX
LPHS
ns
***
B
testis
Ratio testes/body weight (g/g)
C
cecum
Ratio cecum/body weight (g/g)
<0.0001
0.0039
D
Hatching blastocyst
Blastocyst
% embryo development
Young
Mature
Paternal condition
E
Metrics by paternal condition
CON nABX LPHS
Batch 1 2 3 1 2 3 1 2 3
# of blastocyst collected for SmartSeq 32 14 21 32 10 32 15 8 23
# of blastocyst that passed quality threshold to perform bioinformatic analysis 31 14 19 31 10 32 15 7 23
Metrics by paternal condition
Young Mature
Batch 1.1 1.2 2 3 4.1 4.2 1 2 3 4
# of blastocyst collected for SmartSeq 23 24 22 7 6 6 30 23 23 5
# of blastocyst that passed quality threshold to perform bioinformatic analysis 19 21 22 7 6 6 29 21 21 5
F
Paternal condition
Development efficiency (%) of blastocysts collected for smartseq
Batch
G
B6 exspoures
B6 aging
Number of reads per samples
Batch 1
Batch 2
Batch 3
Batch 4
Paternal condition
CON
nABX
LPHS
Young
Mature
H
B6 exposures
B6 aging
Number of embryos
Female
Male
Paternal condition
I
EPI marker geneset log2(CPM)
PrE marker geneset log2(CPM)
p=0.90
p=0.42
p=0.24
p=0.49
J
Pat. nABX
Pat. LPHS
solute sodium symporter activity
sodium ion transporter activity
ATP synthesis
mitochondrial respirasome
oxidative phosphorylation
aerobic respiration
mitochondrial respiratory complex IV
ribosome
cytosolic ribosome
transcription repressor activity
transcription regulator complex
methyl–CpG binding
FVB
B6

 © The Author(s)

Figure EV4. Interrogating the effect of age and genetic background on the IEI response in F1 blastocysts.

(**A**) Line plots showing growth curve of B6 males across CON, nABX and LPHS paternal treatments. Error bars indicate standard deviation at each timepoint. (**B**) Dot and bar plots showing testis/body weight ratio for B6 males after treatment. Error bars indicate standard deviation. (**C**) Dot and bar plots showing caecum/body weight ratio for young B6 males after treatment. Error bars indicate standard deviation. (**D**) Dot and bar plots showing rate of F1 blastocyst development and hatching from B6 across paternal treatment (left) and across ages (right). Error bars indicate standard deviation at each timepoint. Bars represent the mean. (**E**) Tables showing numbers of embryos collected for Smart-seq and passed quality thresholds for Smart-seq bioinformatic analysis from B6 across paternal treatment (top) and across ages (bottom). All batches shown. (**F**) Bar charts showing developmental efficiency of blastocysts collected for Smart-seq across batches from B6 (top) and across ages (bottom). All batches are shown. Each dot represents the efficiency of blastocyst development in each culture well. Boxes represent the mean, and error bars indicate the standard deviation. (**G**) Dot plots showing depth of blastocyst sequencing obtained from young B6 fathers across paternal treatment (left), and across paternal ages (right). All batches are shown. (**H**) Grouped bar chart showing number of male and female blastocysts obtained from young B6 fathers across paternal treatment (left), and across paternal ages (right). All batches are shown. (**I**) Total expression (log_2CPM) of selected epiblast (EPI) (left) and primitive endoderm (PrE) (right) geneset markers. Markers used for the respective geneset (see Fig EV2D). P-values were computed using a two-tailed Wilcoxon test. (**J**) Bubble plot showing gene set enrichment analysis (GSEA) comparing the paternal treatments across FVB and B6 backgrounds. (**A–D**) *P* values were computed using an unpaired t-test; [ns: not significant; **P* value < 0.05; ***P* value < 0.005; ****P* value < 0.0005].

© The Author(s)

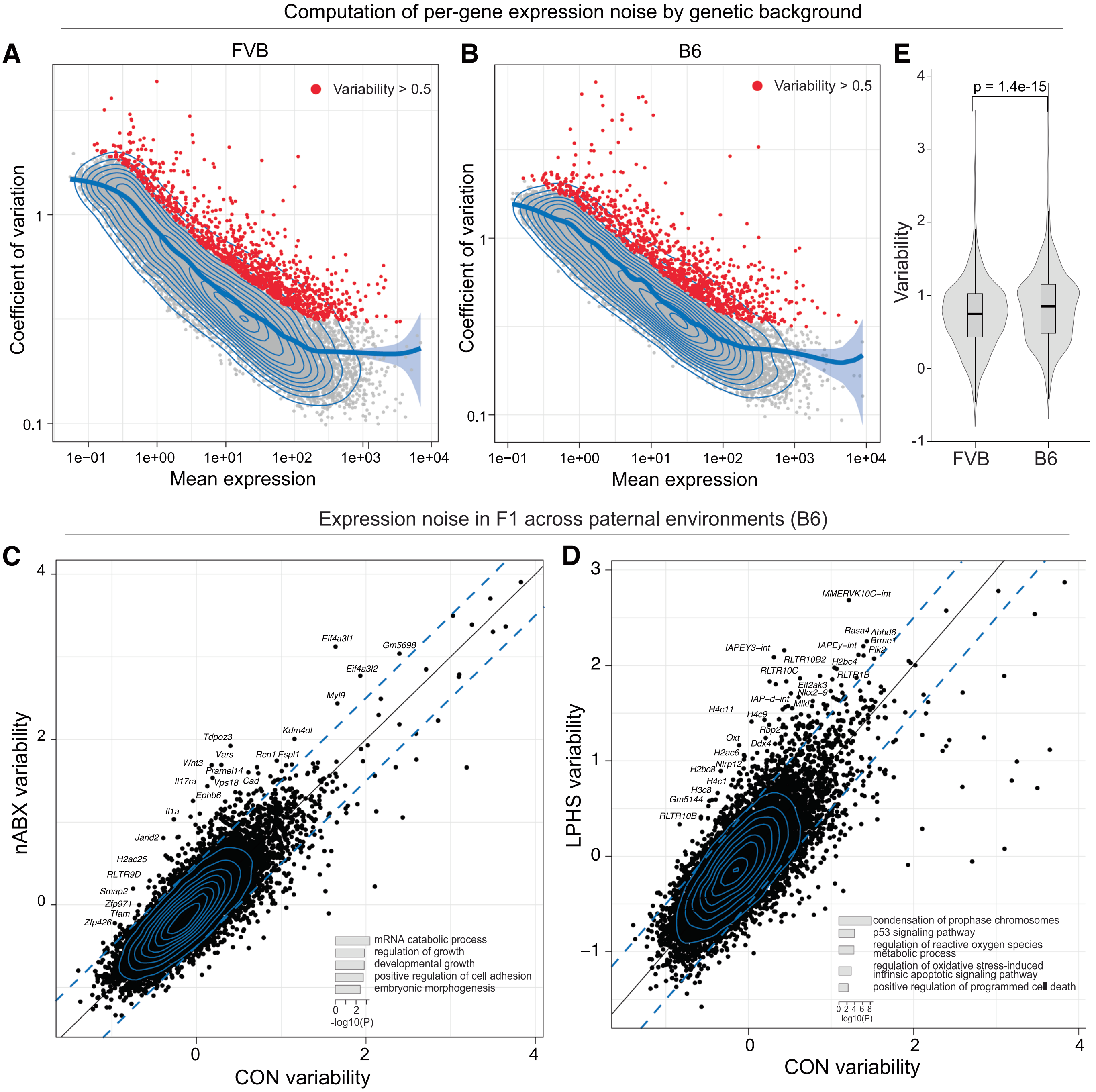

Figure EV5. Gene expression variability across FVB and B6 backgrounds.

(**A**, **B**) Scatter plots plotting coefficient of variation (CV) versus mean expression in FVB (**A**) and B6 (**B**) blastocysts. Blue line represents a generalised additive model (GAM) fit to the CV vs mean expression distribution. Red dots indicated genes with a variability score > 0.5 (see "Methods"). (**C**, **D**) Scatter plots contrasting the variability of genes in the nABX-derived blastocysts (**C**) and LPHS-derived blastocysts (**D**) relative to controls in the B6 background. Genes showing higher variability in blastocysts of treated males are highlighted. Horizontal bar chart highlighting enriched Gene Ontology (GO) terms in genes showing high variability in nABX-derived blastocysts (left) and LPHS-derived blastocysts (right) relative to controls in the B6 background. (**E**) Violin and box plot demonstrating the relative distribution of variability between the FVB and B6 backgrounds. P-value was computed using paired two-tailed *t* test. [#genes = 3738]. [Box plots: horizontal black line at the centre is the median. The box extends from the first quartile (Q1: 25th percentile) to the third quartile (Q3: 75th percentile). The vertical black line extends from Q1 − 1.5 × IQR to Q3 + 1.5 × IQR, where IQR = interquartile range. The minima and maxima are not explicitly annotated in these figures.

 © The Author(s)

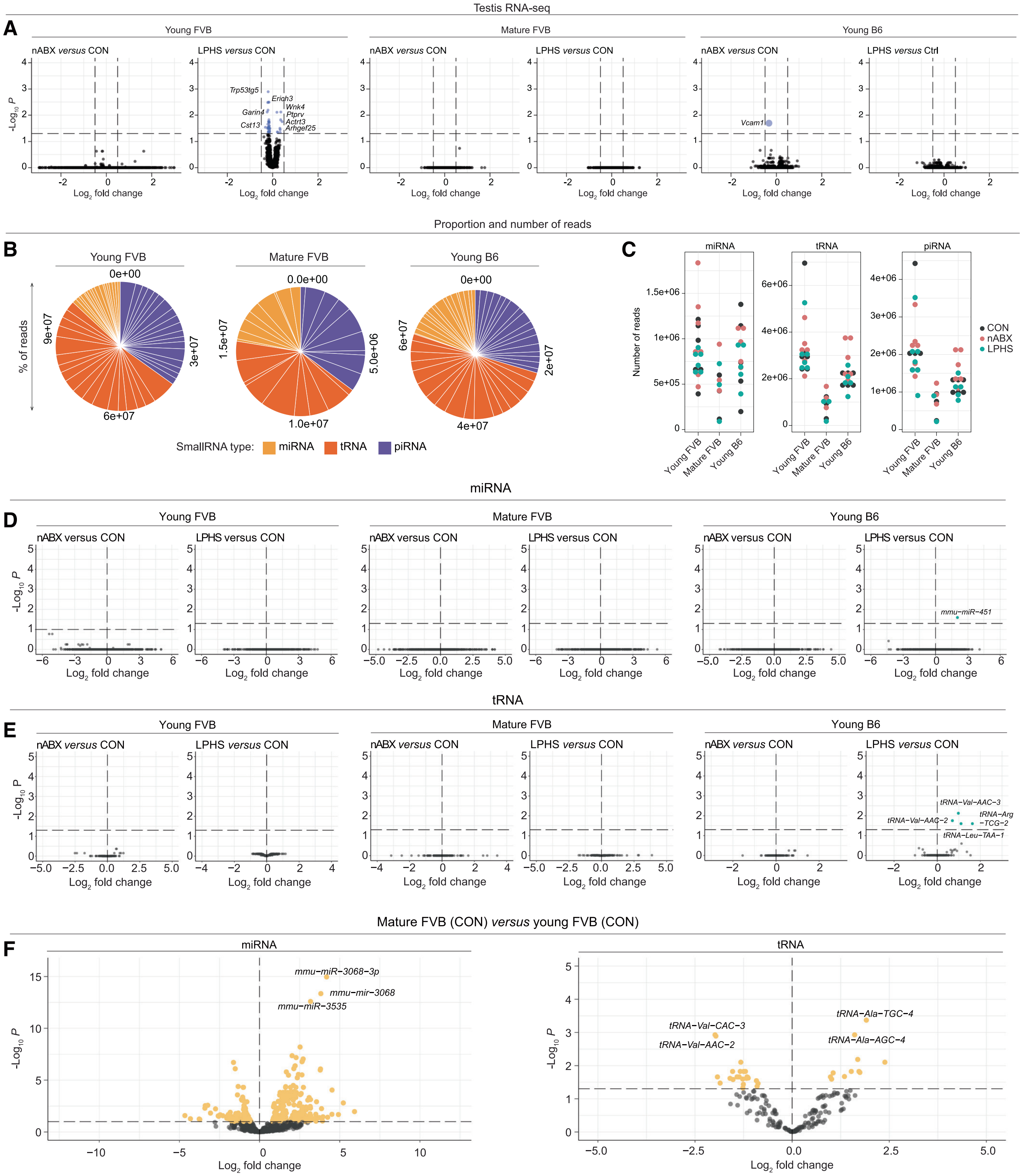
Testis RNA-seq
A
Young FVB
Mature FVB
Young B6
nABX versus CON
LPHS versus CON
LPHS versus Ctrl
Trp53tg5
Eriqh3
Wnk4
Garin4
Ptprv
Actrl3
Cst13
Arhgef25
Vcam1
Log2 fold change
-Log10 P
Proportion and number of reads
B
C
0e+00
9e+07
3e+07
6e+07
0.0e+00
1.5e+07
5.0e+06
1.0e+07
6e+07
2e+07
4e+07
% of reads
SmallRNA type: miRNA tRNA piRNA
miRNA
tRNA
piRNA
Number of reads
CON
nABX
LPHS
Young FVB
Mature FVB
Young B6
miRNA
D
mmu-miR-451
tRNA
E
tRNA-Val-AAC-3
tRNA-Val-AAC-2
tRNA-Arg-TCG-2
tRNA-Leu-TAA-1
Mature FVB (CON) versus young FVB (CON)
F
mmu-miR-3068-3p
mmu-mir-3068
mmu-miR-3535
tRNA-Val-CAC-3
tRNA-Val-AAC-2
tRNA-Ala-TGC-4
tRNA-Ala-AGC-4

Figure EV6. Paternal reproductive response to nABX, LPHS and aging across genetic backgrounds.

(**A**) Volcano plots depicting differential gene expression in testis transcriptomes upon nABX and LPHS treatments in young FVB (left), mature FVB (middle) and young B6 (right) genetic backgrounds. [#genes: YoungFVB=21231, matureFVB=16586, youngB6 = 16529]. (**B**) Pie charts showing relative distributions of different types of small RNAs in sperm of young FVB (left), mature FVB (middle) and young B6 (right) males. (**C**) Dot plots showing depth of small RNA sequencing split into miRNA (left), tRNA (middle) and piRNA (right) reads split across genetic background and coloured by condition. (**D**) Volcano plots of differential expression of miRNAs in sperm of nABX (left) and LPHS (right) males relative to controls in young FVB, mature FVB and young B6 genetic background. [#miRNAs: YoungFVB=1271, matureFVB=1258, youngB6 = 1256]. (**E**) Volcano plots of differential expression of tRNAs in sperm of nABX (left) and LPHS (right) males relative to controls in young FVB, mature FVB and young B6 genetic background. [#tRNAs: YoungFVB=207, matureFVB=224, youngB6 = 198]. (**F**) Volcano plots of differential expression of miRNAs (left) and tRNAs (right) in sperm of untreated (CON) mature FVB versus young FVB males. [#miRNAs=1148, #tRNAs=264]. (**A**, **D**–**F**) All volcano plots plot adjusted *P* values versus $\log_2$ fold-change values. Thresholds for significance are set at adjusted *P* value < 0.05.

© The Author(s)
